# Novel multiplex TaqMan assay for differentiation of the four major pathogenic *Brachyspira* species in swine

**DOI:** 10.1002/mbo3.1169

**Published:** 2021-02-16

**Authors:** Simone Scherrer, Roger Stephan

**Affiliations:** ^1^ Department of Veterinary Bacteriology Institute for Food Safety and Hygiene Vetsuisse Faculty University of Zurich Zurich Switzerland

**Keywords:** *B. hampsonii*, *B. hyodysenteriae*, *B. pilosicoli*, *B. suanatina*, *Brachyspira* species differentiation, LNA‐ and MGB‐probes, TaqMan 5‐plex qPCR

## Abstract

A novel TaqMan 5‐plex real‐time PCR using a combination of locked nucleic acid‐modified (LNA)‐ and minor groove binding (MGB)‐conjugated DNA probes was developed for identification and differentiation between the four main pathogenic *Brachyspira* species in swine. *B*.* hyodysenteriae*, *B*.* pilosicoli*, and *B*.* suanatina* are identified using three hydrolysis probes targeting *cpn60*, while *B*.* hampsonii* is recognized by another *nox* specific probe. The assay also includes an exogenous internal control simultaneously verifying the PCR competency of the DNA samples. Validation of the novel assay was performed using DNA samples from 18 *Brachyspira* reference strains and 477 clinical samples obtained from porcine rectal swabs by comparing them with different PCR‐based methods targeting *nox*, 16S rDNA, and 23S rDNA. The specificity of the assay was 100% without cross‐reactivity or detection of different pathogens. Depending on the *Brachyspira* species, the limit of detection was between 10 and 20 genome equivalents with a cut‐off threshold cycle (Ct) value of 37. The developed highly sensitive and specific 5‐plex real‐time PCR assay is easy to implement in routine veterinary diagnostic laboratories and enables rapid differentiation between the main four pathogenic *Brachyspira* species recognized in pigs using a single‐tube approach.

## 1 INTRODUCTION

The genus *Brachyspira* (*B*.) presently comprises ten species of anaerobic spirochaetes of the large intestines, including *B*. *hyodysenteriae*, *B*. *pilosicoli*, *B*. *hampsonii*, *B*. *suanatina*, *B*. *aalborgi*, *B*. *intermedia*, *B*. *innocens*, *B*. *murdochii*, *B*. *alvinipulli* (Hampson et al., 2019), and one new species isolated from vervet monkeys designated as *B*. *catarrhinii* sp. nov. (Phillips et al., 2019). Diverse mammalian and avian hosts including humans can be inhabited by this genus harboring a wide variability in pathogenic potential. Globally, *B*. *hyodysenteriae* is the most important pathogenic species in pigs responsible for significant economic loss in affected farms causing swine dysentery (Hampson et al., 2015). *B*. *pilosicoli*, which can be encountered in many host species including pigs, humans, poultry, dogs, and horses, is the etiologic agent of porcine intestinal spirochaetosis, an enteric disease‐causing chronic diarrhea and mild colitis (Hampson et al., 2006; Trott et al., 1996). *B*. *intermedia* and *B*. *alvinipulli* are the causing agents of avian intestinal spirochaetosis (McLaren et al., 1997; Stanton et al., 1998). *B*. *innocens* and *B*. *murdochii*, which can be encountered in pigs, chickens, and rats, have not been associated to any disease and are considered as harmless commensals (Stephens & Hampson, 2001). *B*. *aalborgi* is only found in humans and higher primates (Hovind‐Hougen et al., 1982; Munshi et al., 2003). More recently, the emergence of two *Brachyspira* species has been described, which are capable of infecting birds and pigs, namely *B*. *hampsonii* (Chander et al., 2012) and *B*. *suanatina* (Rasback et al., 2007) both harboring strong hemolytic properties with clinical signs indistinguishable from swine dysentery.

Species identification is commonly performed by PCR assays, restriction fragment length polymorphism (RFLP) (Rohde & Habighorst‐Blome, 2012), or by partial NADH oxidase gene (*nox*) sequencing (Atyeo et al., 1999). Most widely used targets for PCR assays detecting *B*. *hyodysenteriae* and/or *B*. *pilosicoli* include *nox* (Atyeo et al., 1999), 16S rDNA (La et al., 2003), 23S rDNA (Borgström et al., 2017; Leser et al., 1997), and *tlyA* (Fellström et al., 2001). It has been shown that some newly emerging *B*. *hampsonii* and *B*. *suanatina* strains may cross‐react or stay undetected in some species‐specific PCRs due to genetic similarities of target genes used for identification of involved strains thereby leading to a misidentification of *B*. *hampsonii* and *B*. *suanatina* (Burrough, 2017; Rohde et al., 2014). The strong hemolytic properties of these strains and the fact of causing a disease indistinguishable from swine dysentery drives the need of developing new routine diagnostic tests to rapidly uncover involved species.

Recently, it has been shown that sequencing of chaperonin *cpn60* is superior to *nox* sequencing and revealed more reliable species identification for some isolates (Rohde et al., 2019). Molecular chaperones are universally present in almost all eubacteria and archaea harboring phylogenetically more discriminative gene sequences for species identification than those of the traditionally used 16S rDNA target (Hill et al., 2004; Links et al., 2012). However, due to massive gene rearrangements within some *Brachyspira* species leading to a diversity of mosaic genomes (Hampson & Wang, 2018) or the presence of a great wealth of *Brachyspira* species (Johnson et al., 2018) it remains a challenge to assign the correct species for a certain minority of isolates independent from the chosen target gene.

To date, no qPCR assay distinguishing simultaneously between the main porcine pathogenic *Brachyspira* strains including *B*. *hyodysenteriae*, *B*. *pilosicoli*, *B*. *hampsonii*, and *B*. *suanatina* in one reaction mixture has been reported. The purpose of the present study was to develop a reliable and robust multiplex qPCR system that can be used to identify and differentiate all pathogenic *Brachyspira* species in swine. To evaluate the novel assay as a diagnostic tool, 503 samples were examined with the novel 5‐plex qPCR and compared to different PCR‐based assays targeting 23S rDNA, *nox*, and 16S rDNA. Given reliable monitoring of *Brachyspira* infections in pigs, it is of great advantage to have an efficient molecular tool for fast and accurate detection of all porcine pathogenic *Brachyspira* species in a one‐tube approach.

## 2 MATERIALS AND METHODS

### 2.1 Brachyspira strains and porcine rectal swabs

18 reference strains representing eight *Brachyspira* species (*B*. *hyodysenteriae*, *B*. *hampsonii*, *B*. *suanatina*, *B*. *pilosicoli*, *B*. *intermedia*, *B*. *innocens*, *B*. *murdochii*, and *B*. *alvinipulli*) were included in the study for the development of the 5‐plex PCR (Table 1). For evaluation purposes, 25 *B*. *hampsonii* isolates received from different laboratories worldwide, one *B*. *suanatina* isolate obtained from a ring trial, and 477 clinical samples from porcine rectal swabs obtained from routine diagnostic submissions to the Department of Veterinary Bacteriology at the Vetsuisse Faculty, University of Zurich, between 2012 and 2020 (Table A1, available at https://doi.org/10.5281/zenodo.4434271) were used. The clinical samples originated from diseased and healthy pigs taken during an active monitoring program on swine dysentery in Switzerland.

**TABLE 1:** 18 *Brachyspira* reference strains used for the development of the novel multiplex qPCR assay

Organism	Strain designation	Result 5‐plex qPCR
*B*. *hampsonii* clade I	ATCC BAA2463	positive in Channel Orange
*B*. *hampsonii* clade II	ATCC BAA2464	positive in Channel Orange
*B. hampsonii*	P280/1^a^	positive in Channel Orange
*B. hampsonii*	5369‐1x/12^b^	positive in Channel Orange
*B. hyodysenteriae*	ATCC 27164	positive in Channel Green
*B. hyodysenteriae*	ATCC 49526	positive in Channel Green
*B. hyodysenteriae*	ATCC 31212	positive in Channel Green
*B. hyodysenteriae*	404/1x/06^b^	positive in Channel Green
*B. suanatina*	ATCC BAA2592	positive in Channel Crimson
*B. pilosicoli*	ATCC 51139	positive in Channel Yellow
*B. pilosicoli*	404/06^b^	positive in Channel Yellow
*B. innocens*	ATCC 29796	Negative
*B. innocens*	8244/05^b^	Negative
*B. murdochii*	ATCC 51284	Negative
*B. murdochii*	403‐2x/06^b^	Negative
*B. intermedia*	ATCC 51140	Negative
*B. intermedia*	863/06^b^	Negative
*B. alvinipulli*	ATCC 51933	Negative

^a^ David Hampson, School of Veterinary and Life Sciences, Murdoch University, Perth, Australia.

^b^ Judith Rohde, Institute for Microbiology, University of Veterinary Medicine, Hannover, Germany.

### 2.2 Culture and identification of clinical samples

Porcine rectal swabs were cultured on selective tryptose soy agar (TSA) and incubated at 42°C in an anaerobic environment (Trilab, Biomerieux, Marcy L’Etoile, France) for 4–6 days as described previously (Borgström et al., 2017; Dünser et al., 1997; Prohaska et al., 2014). Subcultures were performed if spirochetes were found by dark‐field microscopy. The resulting colonies were washed off with 1 ml of ultrapure water and DNA was obtained through thermal lysis by boiling the bacterial cell suspension for 10 min at 99°C with a subsequent centrifugation step at 17,000 *g* for 3 min. 2 μl of the obtained supernatant containing DNA was used as a template in the PCR reaction. The concentration of the obtained DNA samples was in the range of 100–400 ng/μl.

DNA samples were identified by multiplex qPCR targeting 23 s rDNA (Borgström et al., 2017). For further identification, DNA samples of a subset of epidemiologically non‐linked clinical samples originating from different farms were chosen for a genus‐specific PCR using primers targeting the *Brachyspira nox* gene (Rohde et al., 2002). Sequencing of PCR amplicons was performed by Sanger sequencing using the forward primer nox (Bnoxf) and analyzed using NCBI Blast (Table A1, available at https://doi.org/10.5281/zenodo.4434271).

### 2.3 Development of the 5‐plex qPCR

**FIGURE 1:**
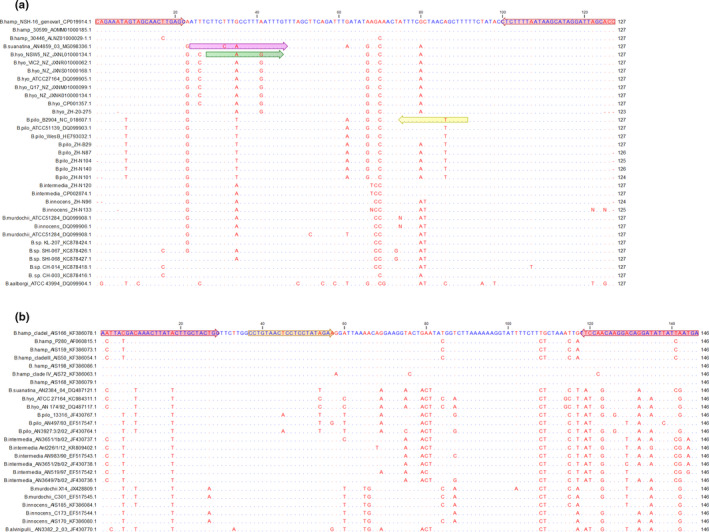
Sequence alignments of amplicons generated in the 5‐plex qPCR assay. Primer sequences are indicated as red arrows. Variable nucleotide positions are highlighted in red, whereas conserved nucleotides are shown in blue. Accession numbers of GenBank of shown sequences are indicated if available. (a) *cpn60*‐amplicon generated using primers cpn60_for and cpn60_rev. The following colored arrows illustrate probe sequences of *Brachyspira* species: pink for *B*. *suanatina*, green for B. *hyodysenteriae*, and yellow for *B*. *pilosicoli* (b) nox‐amplicon generated using primers nox_for and nox_rev. The *B*. *hampsonii*‐specific probe is indicated on the gene *nox* in orange

Primers and probes were designed using CLC Main Workbench software 7.5.1 from alignments of available *cpn60* sequences from the NCBI databank (Rohde et al., 2019). Additionally, *cpn60* of nine clinical samples were partially sequenced (Figure 1). Primers were designed on a conserved region of *cpn60* (cpn60_for: 5′‐ CRGAAATWGTMGCAACYTGAGC −3′ and cpn60_rev: 5′‐ GGYGCWAATCCTATGCTTATTAAAAGAGG −3′) amplifying a 127‐base pair (bp) fragment of *B*. *hyodysenteriae*, *B*. *pilosicoli*, *B*. *hampsonii*, and *B*. *suanatina*. On the 127‐bp amplicon, target sequences for TaqMan probes specific for *B*. *hyodysenteriae*, *B*. *pilosicoli*, and *B*. *suanatina* were chosen. A second primer pair specific for *B*. *hampsonii* was designed on *nox* gene (nox_for: 5′‐TCATTRATRATATCCTGTCCTTGTKGGAA‐3′ and nox_rev: 5′‐AATTACGACAAACTTATACTTGCTACTGG‐3′). All probe sequences are listed in Table 2 with the respective labeled reporter dye. Probes for *B*. *pilosicoli* and *B*. *hyodysenteriae* (Thermo Fisher Scientific, Renfrewshire, UK) comprise minor groove binding (MGB) molecules at the 3′‐end enabling relatively short probe sequences to be species‐specific, thus increasing the specificity of the probes (Kutyavin et al., 2000). Targets for *B*. *suanatina* and *B*. *hampsonii* utilize locked nucleic acid (LNA) probes, also increasing the probe's specificity by allowing the formation of stable hybridization products. Quenching of the LNA probes at the 3′‐end is performed by black hole quencher 3 (BHQ3) in the case of *B*. *suanatina* and QXL610 in the case of *B*. *hampsonii*, both belonging to the group of dark non‐fluorescent quenchers (Eurogentec S.A., Seraing, Belgium).

**TABLE 2:** Sequences of probes and primers used for the TaqMan multiplex qPCR assay. Channels for measuring the different fluorophores calculated PCR efficiencies measured in the linear range, and *r* ^2^ values are indicated. Borgström et al. (2017) represent locked nucleic acid bases (LNA) and MGB stands for minor groove binding probe

*Brachyspira* species	gene	name	Probe / Primer (5′→3′)	Channel	*r* ^2^ value	Efficiency (%)
*B. hyodysenteriae*	*cpn60*	Probe_hyo_MGB	FAM‐CTTCTTTACCTTTGATTTG‐MGB	Green	.998	99
*B. pilosicoli*	*cpn60*	Probe_pilo_MGB	VIC‐AAAGCAGTTAGYGAAAT‐MGB	Yellow	.995	97
*B. suanatina*	*cpn60*	Probe_suana_LNA	AlexaFluor680‐AT{T}TCTTC{C}TT{A}CCTTT{A}ATTTGT‐BHQ‐3	Crimson	.999	99
	*cpn60*	Primer_cpn60_for	CRGAAATWGTMGCAACYTGAGC			
	*cpn60*	Primer_cpn60_rev	GGYGCWAATCCTATGCTTATTAAAAGAG			
*B. hampsonii*	*nox*	Probe_hamp_LNA	Rox‐CCT{G}TAAC{T}CCTCCTAT{A}GAA‐QXL610	Orange	.996	93
	*nox*	Primer_nox_for	AATTACGACAAACTTATACTTGCTACTGG			
	*nox*	Primer_nox_rev	TCATTRATRATATCCTGTCCTTGTKGGAA			

The specificity of both primer and probe sequences were confirmed by BLAST searches. Oligonucleotide primers were synthesized by Microsynth (Balgach, Switzerland).

For monitoring the potentially inhibitory behavior of each PCR reaction, an internal amplification control (IAC) was added to the master mix. Therefore, five femtogram (fg) of a plasmid containing enhanced green fluorescent protein (eGFP) gene was used to generate a 177 bp long amplicon with eGFP‐specific primers eGFP_forward (5′‐GACCACTACCAGCAGAACAC‐3′) and eGFP_reverse (5′‐GAACTCCAGCAGGACCATG‐3′) and detected by the eGFP‐probe (5′‐ATTO 647 N‐AGCACCCAGTCCGCCCTGAGCA‐BHQ3‐3′) (Hoffmann et al., 2006).

All qPCR experiments were performed on a Rotor‐Gene Q (Qiagen) using TaqPath 1‐Step Multiplex Master Mix (Thermo Fisher Scientific). The setup of the Rotor‐Gene instrument included an autogain optimization step for each channel before starting with the first fluorescence acquisition at the beginning of PCR. The total reaction volume was 15 µl. 2 µl of sample DNA was added to a reaction mixture containing TaqPath 1‐Step Multiplex Master Mix, 400 nM of primers targeting *cpn60* and *nox*, 200 nM of primers targeting eGFP, 100 nM of probes hyo_MBG, pilo_MGB, suana_LNA, and hamp_LNA, 25 nM of probe eGFP, 1 µl 5 fg eGFP DNA and ultrapure water. The PCR thermocycling conditions were as follows: initial denaturation at 95 °C for 2 min, 40 cycles with denaturation at 95°C for 15 s, and annealing/extension at 62°C for 60 s. DNA originating from four ATCC reference strains (*B*. *hyodysenteriae* ATCC 27164, *B*. *pilosicoli* ATCC 51139, *B*. *hampsonii* ATCC BAA2463, and *B*. *suanatina* ATCC BAA2592) was used as positive controls in each PCR run. To exclude contaminations in the reaction mixture, ultrapure water was added as a negative control in each experiment.

18 reference strains (Table 1) were used to develop the 5‐plex qPCR assay. The multiplex format was optimized regarding probe and primer concentrations by evaluating different concentration gradients. Data analysis was performed using Rotor‐Gene Q Software 2.3.1 (Qiagen). Samples with a threshold cycle (C_t_) value of ≤37 were considered positive. DNA samples with no detected fluorescent signal for IAC were repeated as 1:5 or 1:10 dilution to minimize potentially inhibitory features.

### 2.4 Specificity

To determine the specificity of the 5‐plex qPCR, an exclusivity panel consisting of 25 pathogenic bacteria was tested (Table A1).

### 2.5 Analytical sensitivity

To determine the analytical sensitivities of the multiplex qPCR, four reference strains (*B*. *hyodysenteriae* ATCC 27164, *B*. *pilosicoli* ATCC 51139, *B*. *suanatina* ATCC BAA 2592, *B*. *hampsonii* ATCC BAA 2463) were examined. Given the genome size of 3.1 Mbp for *B*. *hyodysenteriae* ATCC 27164 (Mirajkar, Johnson, et al., 2016), 2.6 Mbp for *B*. *pilosicoli* ATCC 51139 (Lin et al., 2013), 3.3 Mbp for *B*. *suanatina* ATCC BAA2592 (Mushtaq et al., 2015), and 3.2 Mbp for *B*. *hampsonii* ATCC BAA2463 (Mirajkar, Phillips, et al., 2016) the following DNA quantities corresponded to 1 GE: 3.3 fg for *B*. *hyodysenteriae*, 2.8 fg for *B*. *pilosicoli*, 3.6 fg for *B*. *suanatina* ATCC BAA 2592, and 3.5 fg *B*. *hampsonii*. In order to obtain an accurate limit of detection (LOD) for each target species and identify a reasonable cut‐off Ct value, 20 replicates of each reference strain were analyzed at the following dilutions with a detailed range of concentration in the low range: 100 GE, 50 GE, 20 GE, 10 GE, 5 GE, 1 GE. The LOD was determined as the analyte concentration that produces at a minimum of 95% positive replicates termed as 95% confidence LOD, which was calculated using GenEx software version 7 (MultiD Analyses AB, Goeteborg, Sweden). The fraction of positive replicates versus the concentration represented at a logarithmic scale was plotted using GenEx.

To examine the intra‐ and inter‐assay variability of the novel qPCR assay representing its repeatability, the above mentioned four reference strains were tested using tenfold dilution series in the linear range between 10^7^ and 100 GE. The variability assays were performed in triplicates in three experiments.

### 2.6 Efficiency

To calculate efficiencies of the multiplex qPCR for each target probe, C_t_ values measured in triplicates were plotted against genomic equivalents (GE) in form of standard curves using different dilution series (10^7^–100 GE) for each reference strain (*B*. *hyodysenteriae* ATCC 27164, *B*. *pilosicoli* ATCC 51139, *B*. *suanatina* ATCC BAA 2592, *B*. *hampsonii* ATCC BAA 2463). The PCR efficiency (E) was calculated from the slope (S) of the dilution curve in the linear range between 10^7^ and 100 GE using the following equation: E = (10^1/−S^‐1) × 100.

### 2.7 Evaluation of novel 5‐plex qPCR

DNA samples from 26 different *Brachyspira* isolates and 477 DNA samples obtained from cultures (confirmed as spirochaetes by dark‐field microscopy) of porcine rectal swabs were analyzed and evaluated with four different PCR assays: i) conventional duplex PCR for the identification of *B*. *pilosicoli* and *B*. *hyodysenteriae* targeting *nox* and 16S rDNA, respectively (La et al., 2003), ii) high resolution melting (HRM) assay for the detection of *B*. *hampsonii* on *nox* (Scherrer et al., 2016), iii) multiplex qPCR targeting 23 s rDNA of *B*. *pilosicoli*, *B*. *hyodysenteriae*, and the apathogenic considered triplet (*B*. *intermedia*, *B*. *innocens*, and *B*. *murdochii*) (Borgström et al., 2017), and iv) novel 5‐plex qPCR.

## 3 RESULTS

### 3.1 Conditions of the new 5‐plex qPCR

**FIGURE 2:**
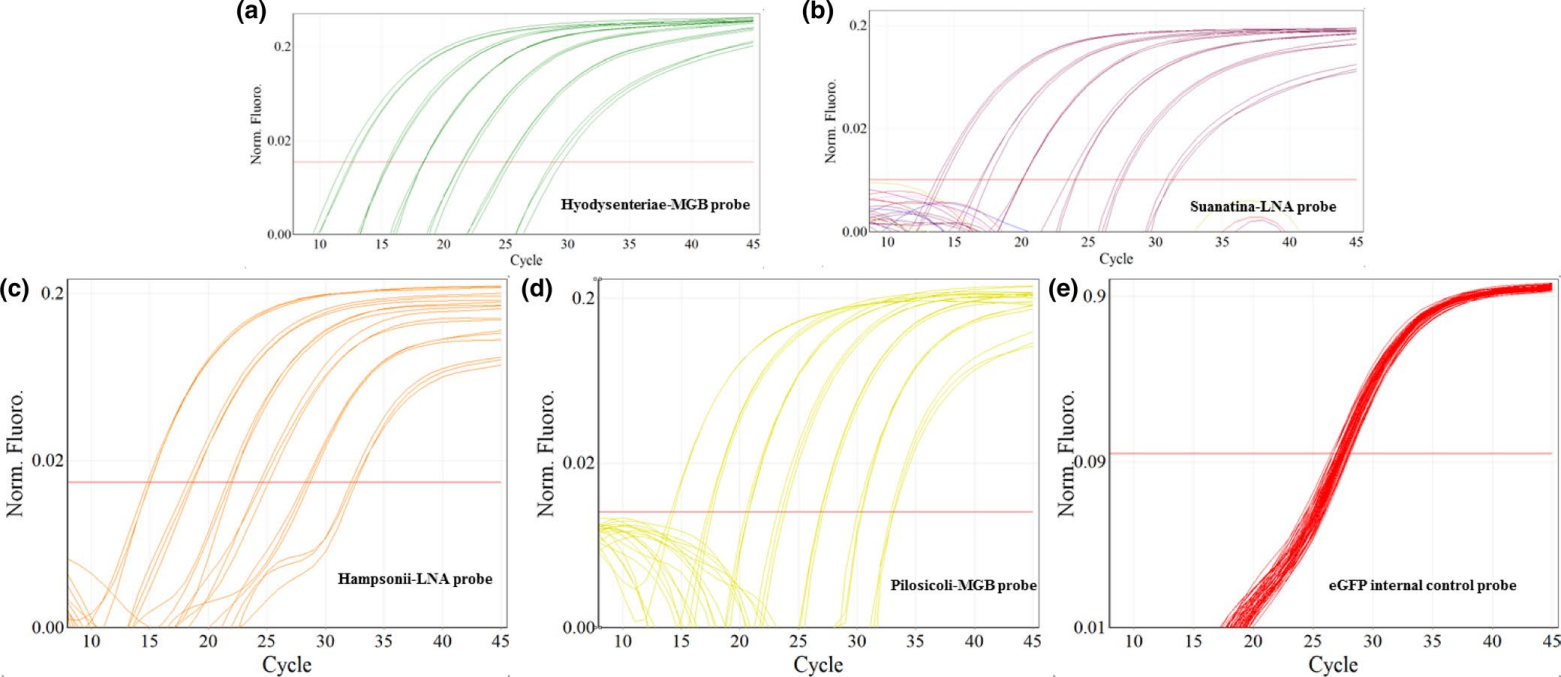
Amplification plots depicting five fluorophores used in the *Brachyspira* 5‐plex qPCR assay. Dilution series obtained from four reference strains (*B*. *hyodysenteriae* ATCC 27164, *B*. *suanatina* ATCC BAA2592, *B*. *hampsonii* ATCC BAA2463, and *B*. *pilosicoli* ATCC 51139) in the linear range of 10^7^–100 genome equivalents representing each fluorophore individually. (a) Channel Green: probe 5’‐ FAM – MGB‐3’ detecting *B*. *hyodysenteriae*, (b) Channel Crimson: probe 5’‐ AlexaFluor680 – BHQ3 ‐3’ detecting *B*. *suanatina*, (c) Channel Orange: probe 5’‐ Rox – QXL610 ‐3’ detecting *B*. *hampsonii*, (d) Channel Yellow: probe 5’‐VIC – MGB ‐3’ detecting *B*. *pilosicoli*, (e) Channel Red: probe 5’‐ CY5 – BHQ1 ‐3’ detecting internal control eGFP

**FIGURE A1:**
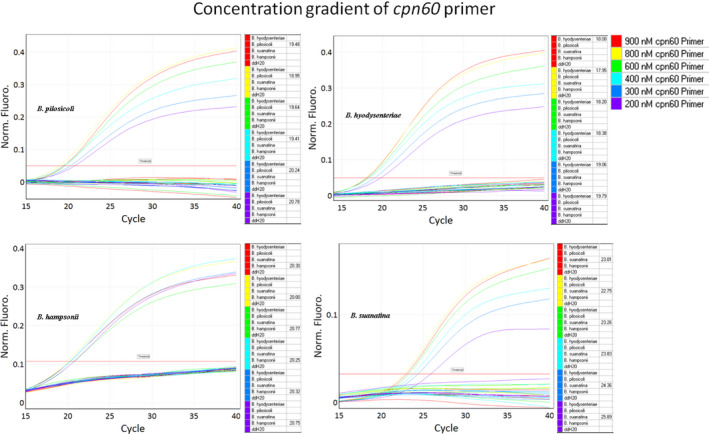
A concentration gradient of *cpn60* primer is illustrated for *B*. *pilosicoli*, *B*. *hyodysenteriae*, *B*. *hampsonii*, and *B*. *suanatina*. A concentration of 400 nM *cpn60* primer is optimal for detection of *B*. *pilosicoli*, *B*. *hyodysenteriae*, and *B*. *suanatina*, while detection of *B*. *hampsonii* with a *nox*‐specific probe is not influenced by a gradient of *cpn60* primer

**FIGURE A2:**
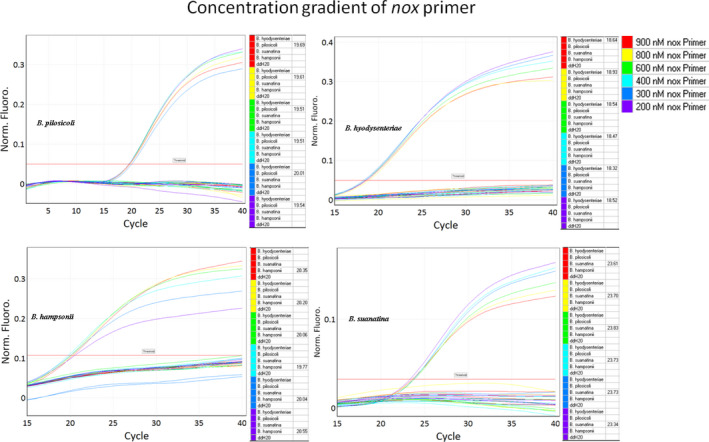
A concentration gradient of *nox* primer is illustrated for *B*. *pilosicoli*, *B*. *hyodysenteriae*, *B*. *hampsonii*, and *B*. *suanatina*. A concentration of 400 nM *nox* primer is optimal for detection of *B*. *hampsonii*, while *cpn60*‐specific detection of *B*. *pilosicoli*, *B*. *hyodysenteriae*, and *B*. *suanatina* is not influenced by a gradient of *nox* primer

**FIGURE A3:**
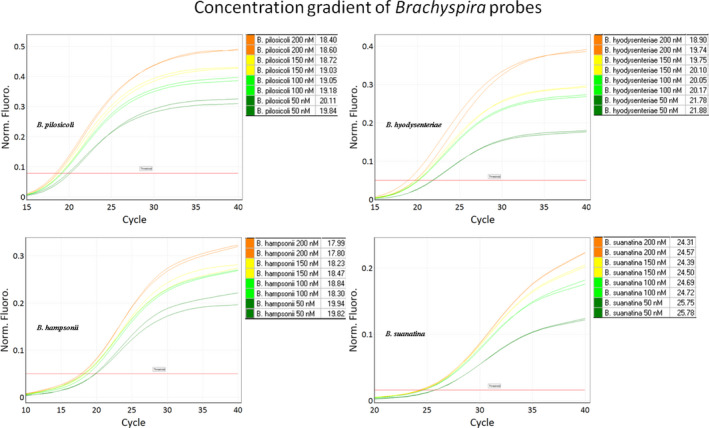
A concentration gradient of *Brachyspira* probes is illustrated for each target. Probe concentrations of 100 nM are optimal for the detection of each target

Optimal primer and concentration gradients were used (Figures A1, A2, A3) to obtain different amplification plots using five distinct detection channels (Figure 2). Channel green, yellow, orange, and crimson can detect *B*. *hyodysenteriae*, *B*. *pilosicoli*, *B*. *hampsonii*, and *B*. *suanatina*, respectively. Furthermore, Channel red can detect eGFP, which acts as internal control proving the conformity of the PCR reaction for correct amplification of the pathogen target.

### 3.2 Specificity

The tested exclusivity panel of 25 pathogenic bacteria resulted in negative results for all strains (Table A1). All reference strains including pathogenic and non‐pathogenic *Brachyspira* spp. examined by qPCR correlated with the expected results (Table 1). Hence, the novel 5‐plex qPCR assay had a specificity of 100%.

### 3.3 Analytical sensitivity

**FIGURE 3:**
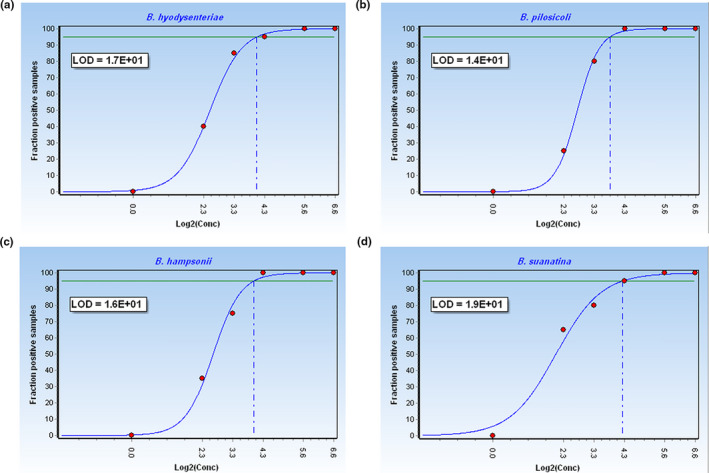
Limit of detection (LOD). Graphs illustrate the fraction of replicate samples with positive reads of dilution series at different concentrations (100 genome equivalents (GE), 50 GE, 20 GE, 10 GE, 5 GE, 1GE) in log scale. LOD is calculated at the relevant confidence level of 95% (green line). Data analysis was performed with GenEx (http://www.multid.se). The cut‐off threshold cycle value was 37. (a) LOD of *B*. *hyodysenteriae*: 17 genome equivalents (GE), (b) LOD of *B*. *pilosicoli*: 14 GE, (c) LOD of *B*. *hampsonii*: 16 GE, and (d) LOD of *B*. *suanatina*: 19 GE

The dynamic range of the standard curve was between 10^7^ and 100 GE for all four tested *Brachyspira* reference strains. The concentration range of the LOD was not part of the linear range and was measured for concentrations <100 GE. The following LODs were identified to be within the relevant confidence level of 95%: 17 GE for *B*. *hyodysenteriae*, 14 GE for *B*. *pilosicoli*, 16 GE for *B*. *hampsonii*, and 19 GE for *B*. *suanatina*, respectively (Figure 3), corresponding to a cut‐off Ct value of 37. The results of the variability assays revealed a variation of CV% of <3% for the inter‐assay variability and <4% for the intra‐assay variability demonstrating the multiplex qPCR to be a highly reproducible and robust assay (Table A2).

### 3.4 Efficiency

**FIGURE 4:**
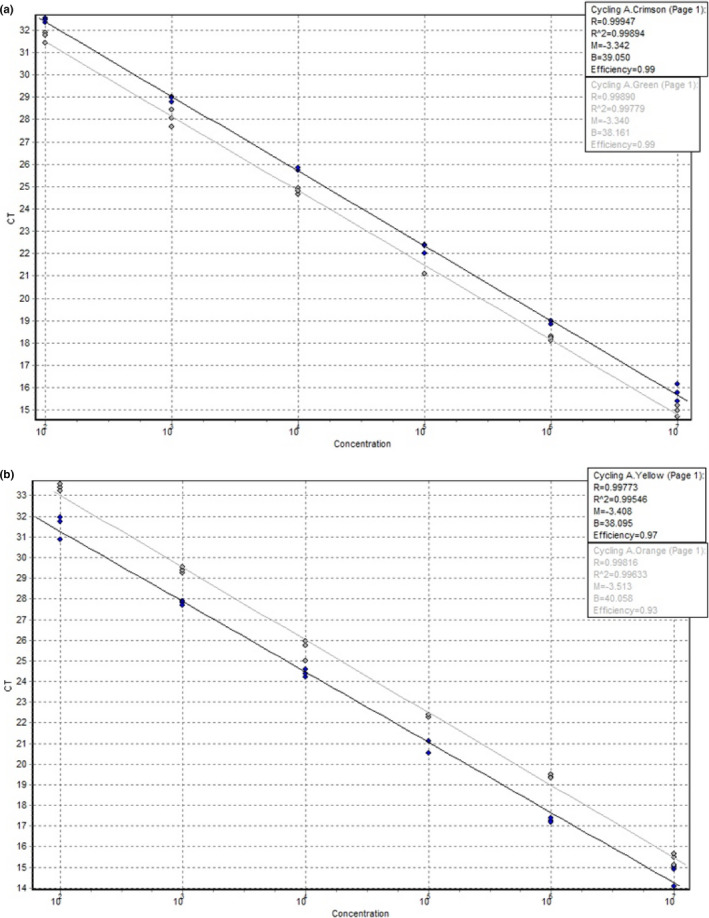
Standard curves of dilution series acquired by the qPCR in the dynamic range of 10^7^ – 100 genome equivalents. (A) *B*. *hyodysenteriae* and *B*. *suanatina* have PCR efficiencies of 99% for both probes. (B) *B*. *pilosicoli* and *B*. *hampsonii* have PCR efficiencies of 97% and 93%, respectively

In the linear range of the tested dilution series between 10^7^ and 100 GE, PCR reactions of each target species resulted in PCR efficiencies of 99%, 99%, 97%, and 93% for *B*. *hyodysenteriae*, *B*. *suanatina*, *B*. *pilosicoli*, and *B*. *hampsonii*, respectively, with correlation coefficient values of >0.995 (Figure 4).

### 3.5 Comparison of the new 5‐plex qPCR with other PCR assays

For evaluation purposes, results obtained from four different PCR assays using DNA samples of 26 different *Brachyspira* isolates and DNA samples of cultures from 477 porcine rectal swabs were compared to results obtained from *nox* sequencing revealing distinct sets of information (Table 3): i) The conventional PCR targeting *nox* and 16S rDNA identified in 11.5% of the porcine swab samples *B*. *hyodysenteriae*, in 22.5% of the samples *B*. *pilosicoli*, in 0.4% of the samples a mixed culture of *B*. *hyodysenteriae* and *B*. *pilosicoli*, and 65.6% of the samples were negative. The conventional PCR could not detect five *B*. *pilosicoli* positive swab samples with less than 100 GE of *B*. *pilosicoli*. Additionally, one sample containing *B*. *pilosicoli* and *B*. *hyodysenteriae* harboring an excess of *B*. *hyodysenteriae*, was identified uniquely as *B*. *hyodysenteriae*, whereas *B*. *pilosicoli* remained undetected by the conventional PCR. ii) Testing the DNA samples from *Brachyspira* isolates and 477 clinical samples with the *B*. *hampsonii* HRM assay identified all 25 (5%) *B*. *hampsonii* isolates correctly, whereas the remaining 477 clinical samples, as well as the B. suanatina isolate, were found to be *B*. *hampsonii* negative. iii) The 23S rDNA qPCR assigned 60.6% of the DNA samples from *Brachyspira* isolates and 477 clinical samples to the group of apathogenic considered *Brachyspira* spp. A total of 12.5% of samples was identified as *B*. *hyodysenteriae* (8.7% *B*. *hyodysenteriae*, 3.2% mixed culture of apathogenic *Brachyspira* spp. and *B*. *hyodysenteriae*, and 0.6% mixed culture of *B*. *hyodysenteriae* and *B*. *pilosicoli*), a total of 23.9% of samples as *B*. *pilosicoli* (9.5% *B*. *pilosicoli*, 13.7% mixed culture of apathogenic *Brachyspira* spp. and *B*. *pilosicoli*, and 0.6% mixed culture of *B*. *hyodysenteriae* and *B*. *pilosicoli*), and 3.6% of samples were negative. One clinical sample (19866–10, Table A1, available at https://doi.org/10.5281/zenodo.4434271), however, resulted in contradicting results by 23S qPCR (*B*. *hyodysenteriae*) compared to the conventional PCR and the novel 5‐plex qPCR (*B*. *pilosicoli*). Sequencing this clinical sample using 23S rDNA, *nox* and *cpn60* revealed a mosaic form of genomic rearrangement (results not shown). Strikingly, the 23S qPCR misidentified all 25 *B*. *hampsonii* isolates. 2 and 14 samples were identified as false‐positive *B*. *hyodysenteriae* and apathogenic triplet, respectively. Moreover, *B*. *suanatina* was also misidentified illustrated by a false‐positive signal for the apathogenic triplet (Table A1). iv) In contrast, the novel 5‐plex qPCR did not yield any false positive or negative results; 59.4% of the DNA samples were found negative thus not harboring any pathogenic *Brachyspira* spp. 24.1% were identified as *B*. *pilosicoli*, 11.9% as *B*. *hyodysenteriae*, 5% as *B*. *hampsonii*, 0.6% as the mixed culture of *B*. *hyodysenteriae* and *B*. *pilosicoli*, and one sample (0.2%) as *B*. *suanatina*.

**TABLE 3:** Comparison of results of testing DNA samples from 26 different *Brachyspira* isolates and 477 clinical samples with four different PCR assays including a conventional PCR targeting at *nox* and 16S rDNA, a high resolution melting (HRM) assay targeting at *nox*, a multiplex qPCR targeting at 23S rDNA, and the newly developed 5‐plex qPCR targeting at *cpn60* and *nox*. The 23S rDNA qPCR revealed false‐positive results for *B*. *hampsonii* (cross‐reaction either with *B*. *hyodysenteriae* or apathogenic probe) and *B*. *suanatina*. The novel 5‐plex qPCR can detect all four pathogenic *Brachyspira* species correctly

	*nox*/16S rDNA conventional PCR La et al., 2003	*nox B*. *hampsonii* HRM Scherrer et al., 2016	23S rDNA qPCR Borgström et al., 2017	*cpn60*/*nox* 5‐plex qPCR this study
*B. hyodysenteriae*	58 (11.53%)	–	44 (8.75%) 2^b^	57 (11.33%)
*B. pilosicoli*	113 (22.46%)	–	48 (9.54%)	118 (23.46%)
*B. hampsonii*	–	25 (4.97%)	–	25 (4.97%)
*B. suanatina*	–	–	–	1 (0.2%)
apathogenic^a^	–	–	305 (60.64%) 15^b^	–
Mixes	–	–	–	–
*B*. *hyo* + apathogenic^a^	–	–	16 (3.18%)	–
*B*. *pilo* + apathogenic^a^	–	–	69 (13.72%)	–
*B*. *pilo* + *B*. *hyo*	2 (0.4%)	–	3(0.59%)	3 (0.6%)
Negative	330 (65.61%)	478 (95.03%)	18 (3.58%)	299 (59.44%)
Total DNA samples	503	503	503	503

^a^ apathogenic indicates the identification of either *B*. *intermedia*, *B*. *innocens* or *B*. *murdochii*.

^b^ false‐positive results due to the cross‐reaction of *B*. *hampsonii* and *B*. *suanatina*.

## 4 DISCUSSION

Comparing the four assays tested, the 5‐plex PCR demonstrated a specificity and sensitivity of 100% for all four target *Brachyspira* species. Considering *B*. *hyodysenteriae*, the conventional PCR (La et al., 2003) demonstrated a specificity and sensitivity of 100%, whereas the 23S qPCR (Borgström et al., 2017) gave rise to false‐positive *B*. *hyodysenteriae* results for two *B*. *hampsonii* isolates resulting in a specificity of 99.5% with a sensitivity of 100%. The 23S qPCR and conventional PCR both were 100% specific for *B*. *pilosicoli*, however, due to a higher detection limit of the conventional PCR, its sensitivity only reached 96% in contrast to a 100% sensitivity of the 23S qPCR. Finally, the *B*. *hampsonii* HRM (Scherrer et al., 2016) was 100% specific and sensitive for *B*. *hampsonii*. A clear advantage of the novel 5‐plex PCR is the ability to reliably identify all four relevant pathogen *Brachyspira* spp. in a one‐tube approach.

Also worth mentioning, is the robust capacity of the 5‐plex PCR to test whole‐cell lysates obtained from selective anaerobic culture using a thermal lysis step. No dilution or further treatment of the obtained DNA samples was necessary since PCR performance was surveilled by a simultaneously added exogenous internal control (eGFP). In rare cases of qPCR inhibition or unusual high background of amplification curves, the DNA samples were diluted 1:5.

In the present study, only one *B*. *suanatina* strain from a *Brachyspira* ring trial was available for the validation assay. The result highlighted the specificity of the *B*. *suanatina* probe, however, more diagnostic samples should be tested in the future for further validation. Moreover, new emerging mosaic genomes of *Brachyspira* might result in the need for further adjustments of the molecular diagnostic assay conditions to continuously ensure reliable identification of all pathogenic *Brachyspira* species.

## 5 CONCLUSION

To conclude, the developed highly sensitive and specific multiplex qPCR assay distinguishing between *B*. *hyodysenteriae*, *B*. *pilosicoli*, *B*. *suanatina*, and *B*. *hampsonii* provides a useful diagnostic tool. The benefits of the robust 5‐plex qPCR are cost‐saving with fewer reactions and time‐saving allowing an enhanced throughput of samples. The implication of this optimized 5‐plex qPCR system in the course of routine veterinary diagnostic laboratories sets a cornerstone for a broad and reliable surveillance strategy of *Brachyspira* infection in pig herds.

## CONFLICT OF INTEREST

None declared.

## AUTHOR CONTRIBUTIONS

**Simone Scherrer:** Conceptualization (equal); Data curation (equal); Investigation (lead); Methodology (lead); Writing‐original draft (lead). **Roger Stephan:** Conceptualization (equal); Data curation (equal); Writing‐original draft (supporting); Writing‐review & editing (lead).

## ETHICS STATEMENT

None required.

## Data Availability

All data relevant to the study are included in the article and the appendices except for the supplementary data which are available in the Zenodo repository at https://doi.org/10.5281/zenodo.4434271 [Table A1: DNA samples from different *Brachyspira* isolates and 477 clinical samples used for validation of the novel 5‐plex qPCR assay, *nox* sequencing results, and comparison of results obtained by four independent PCR assays].

## APPENDIX A1

**TABLE A1:** Exclusivity panel of 25 bacterial isolates used for specificity testing of the 5‐plex qPCR

Organism	Source/Strain	Result pentaplex qPCR
*Borrelia burgdorferi*	ATCC 35210	negative
*Borrelia heimsii*	ATCC 35209	negative
*Klebsiella pneumoniae*	clinical isolate^a^	negative
*Trueperella pyogenes*	ATCC 19411	negative
*Streptococcus equi* spp. *equi*	clinical isolate^a^	negative
*Streptococcus equi* spp. suis	clinical isolate^a^	negative
*Pasteurella multocida*	clinical isolate^a^	negative
*Aeromonas hydrophila*	ATCC 35654	negative
*Campylobacter coli*	ATCC 33559	negative
*Campylobacter jejuni*	ATCC 33560	negative
*Clostridium perfringens*	ATCC 13124	negative
*Pseudomonas aeruginosa*	ATCC 27853	negative
*Staphylococcus aureus*	ATCC 25923	negative
*Enterococcus faecalis*	ATCC 29212	negative
*Escherichia coli*	clinical isolate^a^	negative
*Staphylococcus aureus*	ATCC 43300	negative
*Enterococcus faecalis*	ATCC 51299	negative
*Corynebacterium pseudotuberculosis*	ATCC 19410	negative
*Staphylococcus intermedius*	ATCC 29663	negative
*Actinobacillus pleuropneumoniae*	ATCC 27088	negative
*Haemophilus parasuis*	ATCC 19417	negative
*Rhodococcus hoagii*	ATCC 25729	negative
*Streptococcus agalactiae*	ATCC 13813	negative
*Corynebacterium renale*	ATCC 19412	negative
*Bordetella bronchiseptica*	clinical isolate^a^	negative

^a^ Strain collection from the Department of Veterinary Bacteriology, University of Zurich, Switzerland.

**TABLE A2:** Inter‐ and intra‐assay variability of reference strains *B*. *hyodysenteriae*, *B*. *suanatina*, *B*. *pilosicoli*, and *B*. *hampsonii*

Isolates			Intra‐assay variability				Intra‐assay variability				Intra‐assay variability				Inter‐assay variability		
*Brachyspira* species	strain	DNA (Genome equivalents)	Ct	Mean Ct	SD	**CV%**	Ct	Mean Ct	SD	**CV%**	Ct	Mean Ct	SD	**CV%**	Mean Ct	SD	**CV%**
*B. pilosicoli*	ATCC 51139	10^7	14.90	15.21	0.45	2.98	15.10	15.18	0.08	0.50	14.42	15.01	0.51	3.42	15.13	0.11	**0.73**
			15.73				15.25				15.23						
			15.00				15.19				15.37						
*B. pilosicoli*	ATCC 51139	10^6	18.01	18.04	0.14	0.79	18.19	18.39	0.24	1.28	17.58	17.59	0.10	0.57	18.01	0.40	**2.24**
			17.92				18.65				17.49						
			18.20				18.33				17.69						
*B. pilosicoli*	ATCC 51139	10^5	21.31	21.27	0.11	0.52	21.66	21.71	0.45	2.06	21.36	21.20	0.32	1.50	21.39	0.28	**1.29**
			21.15				22.18				21.4						
			21.36				21.29				20.83						
*B. pilosicoli*	ATCC 51139	10^4	24.43	24.16	0.29	1.18	24.67	24.97	0.32	1.29	24.84	24.67	0.17	0.69	24.60	0.41	**1.67**
			23.86				24.93				24.5						
			24.18				25.31				24.68						
*B. pilosicoli*	ATCC 51139	10^3	27.56	27.60	0.13	0.47	27.75	27.75	0.04	0.14	28.07	28.06	0.10	0.36	27.80	0.24	**0.86**
			27.74				27.71				28.16						
			27.49				27.79				27.96						
*B. pilosicoli*	ATCC 51139	10^2	30.85	30.82	0.15	0.48	31.24	31.07	0.18	0.56	31.99	31.79	0.10	0.32	31.22	0.50	**1.61**
			30.95				30.89				31.14						
			30.66				31.07				32.23						
*B. pilosicoli*	ATCC 51139	50	32.19	31.74	0.40	1.26	31.51	32.11	0.64	1.98	32.16	32.56	0.57	1.76	32.14	0.41	**1.27**
			31.61				32.78				32.86						
			31.42				32.05				32.65						
*B. pilosicoli*	ATCC 51139	20	33.57	33.35	0.24	0.72	33.82	33.71	0.78	2.30	33.2	33.34	0.36	1.08	33.47	0.21	**0.62**
			33.09				34.42				33.82						
			33.38				32.88				33.01						
*B. pilosicoli*	ATCC 51139	10	33.50	33.68	0.18	0.52	35.05	34.66	0.57	1.65	35.22	35.45	0.48	1.34	34.60	0.89	**2.57**
			33.69				34.92				36						
			33.85				34.00				35.14						
*B. pilosicoli*	ATCC 51140	5	34.46	36.37	1.65	4.55	39.40	39.55	0.21	0.52	36.94	38.01	1.08	2.84	37.97	1.59	**4.19**
			37.41				39.69				37.99						
			37.23				‐				39.1						
*B. hyodysenteriae*	ATCC 27164	10^7	13.57	13.41	0.29	2.13	14.08	14.03	0.17	1.22	14.02	13.62	0.39	2.83	13.69	0.31	**2.30**
			13.58				14.17				13.25						
			13.08				13.84				13.60						
*B. hyodysenteriae*	ATCC 27164	10^6	16.59	16.48	0.10	0.61	17.09	17.23	0.19	1.09	16.95	16.92	0.17	0.99	16.88	0.37	**2.21**
			16.39				17.44				16.74						
			16.47				17.15				17.07						
*B. hyodysenteriae*	ATCC 27164	10^5	19.30	19.45	0.13	0.68	20.37	20.28	0.20	0.96	20.08	20.01	0.08	0.42	19.91	0.43	**2.14**
			19.56				20.06				19.92						
			19.48				20.42				20.04						
*B. hyodysenteriae*	ATCC 27164	10^4	24.34	24.25	0.23	0.93	24.03	23.82	0.26	1.08	24.19	24.13	0.08	0.34	24.07	0.22	**0.93**
			23.99				23.53				24.17						
			24.41				23.89				24.04						
*B. hyodysenteriae*	ATCC 27164	10^3	27.29	27.47	0.29	1.05	27.22	27.32	0.25	0.90	27.19	27.33	0.26	0.96	27.37	0.08	**0.30**
			27.80				27.14				27.63						
			27.31				27.60				27.16						
*B. hyodysenteriae*	ATCC 27164	10^2	29.81	30.18	0.46	1.54	30.41	30.60	0.19	0.62	31.50	30.63	0.90	2.93	30.47	0.25	**0.83**
			30.70				30.79				30.68						
			30.03				30.61				29.71						
*B. hyodysenteriae*	ATCC 27164	50	33.00	32.26	0.65	2.01	31.58	31.71	0.46	1.46	31.58	32.26	1.13	3.51	32.08	0.32	**0.99**
			31.98				32.23				31.64						
			31.80				31.33				33.57						
*B. hyodysenteriae*	ATCC 27164	20	32.47	32.78	0.27	0.82	33.91	34.32	1.14	3.33	32.63	32.40	0.35	1.07	33.17	1.02	**3.07**
			32.92				33.44				32.56						
			32.95				35.61				32.00						
*B. hyodysenteriae*	ATCC 27164	10	32.16	34.20	1.93	5.66	36.89	35.63	1.78	5.00	35.45	34.63	1.32	3.82	34.82	0.74	**2.11**
			34.42				34.37				33.10						
			36.01								35.33						
*B. hyodysenteriae*	ATCC 27164	5	33.67	34.44	0.90	2.62	39.03	38.48	1.55	4.02	33.08	33.08	‐	‐	35.33	2.81	**7.95**
			35.43				39.67				‐						
			34.21				36.73				‐						
*B. suanatina*	ATCC BAA 2592	10^7	14.69	14.61	0.08	0.55	13.62	13.95	0.30	2.17	14.04	13.99	0.04	0.30	14.19	0.37	**2.60**
			14.61				14.21				13.96						
			14.53				14.03				13.98						
*B. suanatina*	ATCC BAA 2592	10^6	16.76	16.64	0.21	1.28	16.88	17.09	0.19	1.08	16.61	16.68	0.08	0.50	16.80	0.25	**1.49**
			16.76				17.16				16.77						
			16.39				17.23				16.65						
*B. suanatina*	ATCC BAA 2592	10^5	19.89	20.11	0.19	0.93	20.36	20.42	0.06	0.28	20.27	20.23	0.04	0.19	20.25	0.16	**0.79**
			20.21				20.44				20.20						
			20.22				20.47				20.21						
*B. suanatina*	ATCC BAA 2592	10^4	23.58	23.61	0.03	0.13	24.05	24.10	0.11	0.45	23.87	23.75	0.16	0.66	23.82	0.25	**1.05**
			23.64				24.02				23.57						
			23.61				24.22				23.80						
*B. suanatina*	ATCC BAA 2592	10^3	27.64	27.50	0.16	0.57	26.99	27.61	0.56	2.02	27.98	27.73	0.22	0.81	27.61	0.12	**0.42**
			27.53				27.75				27.54						
			27.33				28.08				27.68						
*B. suanatina*	ATCC BAA 2592	10^2	30.30	29.81	0.46	1.54	30.13	30.13	0.08	0.27	30.63	30.15	0.42	1.39	30.03	0.19	**0.63**
			29.39				30.21				29.88						
			29.75				30.05				29.93						
*B. suanatina*	ATCC BAA 2592	50	31.19	31.97	0.71	2.23	30.97	31.51	0.47	1.48	31.72	31.56	0.25	0.81	31.68	0.25	**0.80**
			32.15				31.78				31.27						
			32.58				31.78				31.70						
*B. suanatina*	ATCC BAA 2592	20	32.38	31.90	0.72	2.27	32.56	32.89	0.71	2.16	32.22	32.23	0.26	0.79	32.34	0.50	**1.55**
			31.07				32.40				32.49						
			32.26				33.70				31.98						
*B. suanatina*	ATCC BAA 2592	10	32.61	32.30	0.29	0.90	32.98	32.84	0.84	2.57	33.30	32.89	0.48	1.46	32.68	0.32	**0.99**
			32.03				33.60				33.01						
			32.27				31.93				32.36						
*B. suanatina*	ATCC BAA 2593	5	31.96	31.33	0.56	1.80	33.55	33.92	0.52	1.52	33.90	35.28	1.95	5.53	33.51	2.01	**5.99**
			31.14				34.28				36.66						
			30.88				‐				‐						
*B. hampsonii*	ATCC BAA2463	10^7	14.26	14.13	0.24	1.72	14.67	14.60	0.14	0.95	13.94	14.23	0.53	3.71	14.32	0.25	**1.73**
			14.28				14.44				13.91						
			13.85				14.69				14.84						
*B. hampsonii*	ATCC BAA2463	10^6	17.69	17.91	0.30	1.67	17.80	18.08	0.26	1.44	18.05	18.38	0.40	2.16	18.12	0.24	**1.31**
			17.79				18.14				18.27						
			18.25				18.31				18.82						
*B. hampsonii*	ATCC BAA2463	10^5	21.29	21.30	0.42	1.97	21.69	21.47	0.24	1.10	21.56	21.73	0.15	0.71	21.50	0.22	**1.00**
			21.73				21.50				21.77						
			20.89				21.22				21.86						
*B. hampsonii*	ATCC BAA2463	10^4	24.20	24.31	0.17	0.68	24.16	24.27	0.39	1.62	25.12	25.11	0.02	0.08	24.57	0.47	**1.93**
			24.23				23.95				25.13						
			24.50				24.71				25.09						
*B. hampsonii*	ATCC BAA2463	10^3	27.49	27.55	0.38	1.39	27.79	28.01	0.23	0.81	28.48	28.31	0.28	0.99	27.96	0.38	**1.37**
			27.96				27.99				28.47						
			27.20				28.24				27.99						
*B. hampsonii*	ATCC BAA2463	10^2	31.10	31.42	0.29	0.92	32.23	32.00	0.23	0.70	31.05	31.01	0.05	0.15	31.48	0.50	**1.59**
			31.66				31.78				31.02						
			31.50				32.00				30.96						
*B. hampsonii*	ATCC BAA2463	50	32.88	33.21	0.29	0.87	32.38	33.01	0.63	1.89	32.74	32.67	0.64	1.95	32.96	0.27	**0.83**
			33.42				33.63				33.27						
			33.33				33.03				32.00						
*B. hampsonii*	ATCC BAA2463	20	35.00	34.52	0.81	2.35	33.28	33.98	0.80	2.35	33.40	34.16	1.29	3.77	34.22	0.27	**0.80**
			33.58				34.85				35.65						
			34.97				33.81				33.44						
*B. hampsonii*	ATCC BAA2463	10	35.72	36.39	1.09	2.99	34.59	35.16	1.16	3.29	38.04	35.79	2.06	5.74	35.78	0.61	**1.71**
			37.64				34.40				34.01						
			35.80				36.49				35.32						
*B. hampsonii*	ATCC BAA2464	5	36.45	36.55	0.14	0.39	34.63	34.97	0.47	1.35	34.33	38.37	5.71	14.87	36.63	1.70	**4.64**
			36.65				35.30				‐						
			‐				‐				42.40						
